# Hemiarthroplasty through SuperPATH versus hemiarthroplasty through conventional approaches in patients with femoral neck fractures: a systematic review and meta-analysis of randomized controlled trials

**DOI:** 10.1038/s41598-023-50206-0

**Published:** 2023-12-21

**Authors:** Nikolai Ramadanov, Katarzyna Jóźwiak, Polina Marinova-Kichikova, Philip Lazaru, Dobromir Dimitrov

**Affiliations:** 1 Center of Orthopaedics and Traumatology, Brandenburg Medical School Theodor Fontane, University Hospital Brandenburg an der Havel, Neuruppin, Germany; 2 Faculty of Health Science Brandenburg, Brandenburg Medical School Theodor Fontane, Brandenburg an der Havel, Germany; 3 Institute of Biostatistics and Registry Research, Brandenburg Medical School Theodor Fontane, Neuruppin, Germany; 4 Department of Surgical Diseases, Faculty of Medicine, Medical University of Pleven, Pleven, Bulgaria; 5 General and Visceral Surgery, Minimally Invasive Surgery and Coloproctology, St. Marien Hospital, Berlin, Germany; 6 Department of Surgical Propedeutics, Faculty of Medicine, Medical University of Pleven, Pleven, Bulgaria

**Keywords:** Health care, Fracture repair, Geriatrics

## Abstract

The aim was to conduct a systematic review of literature and meta-analysis of randomized controlled trials (RCTs) comparing short-term outcomes of bipolar hemiarthroplasty (HA) through SuperPATH and bipolar HA through conventional approaches (CAs) in patients with femoral neck fractures. The following PICO question was formulated: In human participants with femoral neck fractures, are the short-term outcomes of SuperPATH HA better than the short-term outcomes of CAs HA? The following databases were searched until 25 August 2023: PubMed, CNKI, CENTRAL of The Cochrane Library, Clinical trials, and Google Scholar. Quality assessment of the RCTs was performed, according to the Cochrane’s Risk of Bias 2 tool and the recommendations of the GRADE system. Furthermore, we evaluated publication bias with funnel plots. Mean differences (MDs) with 95% confidence intervals (CIs) were calculated for continuous variables using the Hartung–Knapp–Sidik–Jonkman method and a random effects model. Nine RCTs with overall 762 patients were included in this meta-analysis. All 9 RCTs were rated with a moderate risk of bias. The quality of evidence of the outcome parameters was rated moderate to very low. The funnel plots were overall broadly symmetrical, possibly indicating low to moderate publication bias. SuperPATH had a longer operation time compared to CAs (MD = 21.79, 95% CI 12.57 to 31.02). SuperPATH decreased incision length (MD = − 4.50; 95% CI − 5.80 to − 3.20), intraoperative blood loss (MD = − 103.96, 95% CI − 150.27 to − 55.66), postoperative drainage volume (MD = − 137.30, 95% CI − 178.74 to − 95.86), time to mobilization (MD = − 3.86; 95% CI − 5.96 to − 1.76), pain VAS ≤ 1 week postoperatively (MD = − 1.81; 95% CI − 2.17 to − 1.45), and hospitalization time (MD = − 4.05; 95% CI − 4.96 to − 3.15). SuperPATH improved HHS ≤ 1 week postoperatively (MD = 11.10; 95% CI 1.65 to 20.54) and HHS 3 months postoperatively (MD = 6.33; 95% CI 3.97 to 8.69). There was no difference in pain VAS 1–3 months postoperatively (MD = − 0.08; 95% CI − 0.22 to 0.05) and HHS 6 months postoperatively (MD = 0.44; 95% CI − 0.11 to 1.00). This is the first meta-analysis comparing SuperPATH HA with CAs HA in patients with femoral neck fractures. SuperPATH HA was superior in the early short-term functional outcome (HHS) compared to CAs HA, reaching minimal clinically important differences. Furthermore, SuperPATH HA showed significantly better results in incision length, blood loss, time to mobilization, pain intensity (VAS), and hospitalization time than CAs HA.

## Introduction

The possible surgical treatment of femoral neck fractures is summarized in two groups: (1) the head-preserving osteosynthesis such as cannulated screw fixation and the dynamic hip screw (DHS), (2) the endoprosthetic hip joint replacement as a total hip arthroplasty (THA) or a bipolar hemiarthroplasty (HA). There is no agreement in the specialist literature as to which surgical procedure represents the gold standard in the surgical treatment of femoral neck fractures^1,2^. Only a decision framework is given, in which the surgeon must select the best operative procedure in each individual patient case, taking into account numerous influencing factors (fracture classification, patient age, bone quality, functional requirements of the patient, general condition, and compliance of the patient, duration from trauma to surgery, manifestation of osteoarthritis)^2^. Nevertheless, hip arthroplasty is undoubtedly indicated in elderly patients with a displaced femoral neck fracture (Garden III-IV), osteoporosis, osteoarthritis, and a duration from fracture to surgery of more than 24 h^1,2^. And specifically, bipolar HA is indicated in elderly patients with a shorter life expectancy of < 5 years, a lower functional requirement, and a lower level of activity^2^. Less frequently, procedures such as unipolar HA or conservative approaches are used, especially in advanced dementia patients with limited mobility and no appreciable quality of life.

**Figure 1:**
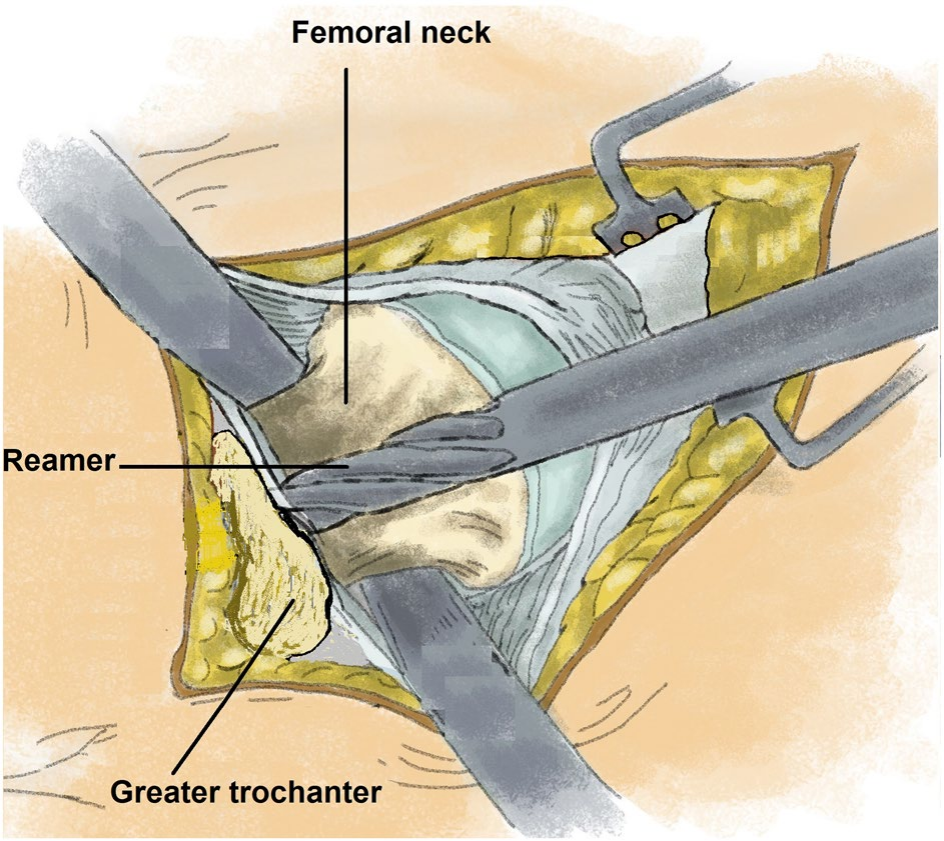
Illustration of the in-situ opening of the femoral canal with a reamer.

**Figure 2:**
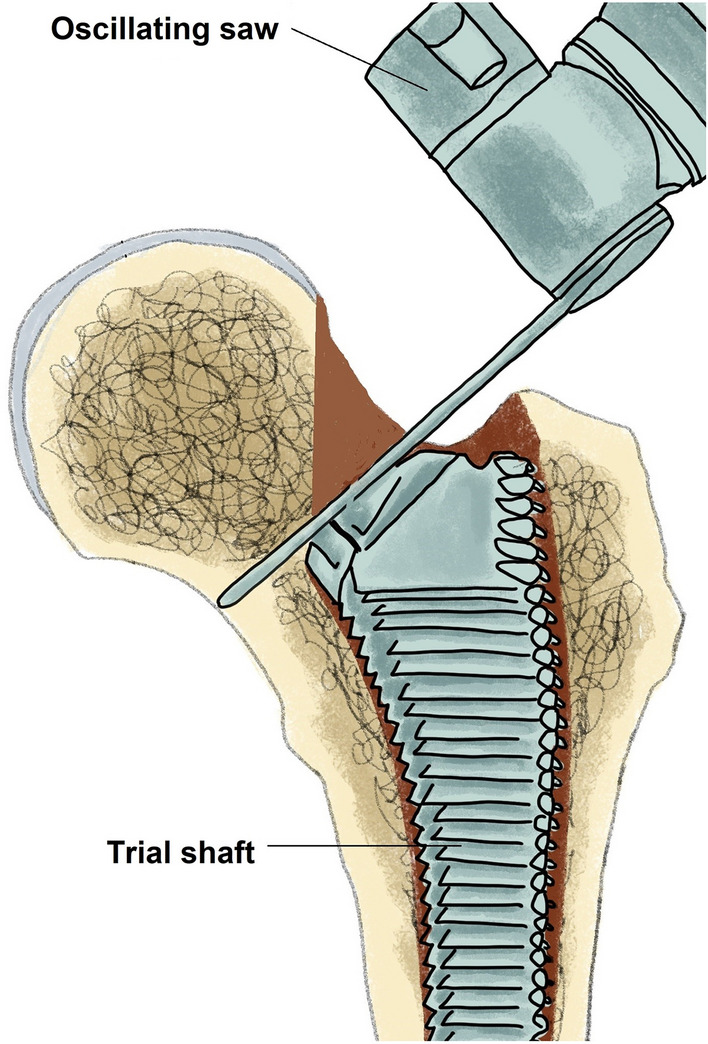
Illustration of the in-situ resection of the femoral neck—a surgical step that characterises the SuperPATH surgical technique.

Hip arthroplasty is one of the most effective and successful operations performed in orthopedic surgery^3^. Continued efforts led to a steady improvement in outcomes after hip arthroplasty over the past century. The further development of the implants, the accumulation of experience by the operating surgeons, the perioperative application of tranexamic acid, and the intraoperative warming of the patient should be mentioned as examples. Furthermore, the improvement in treatment was achieved through the invention and introduction of novel operational approaches and surgical techniques. As part of this endeavor, James Chow introduced a new type of minimally invasive hip approach in 2011 – the Supercapsular percutaneously assisted approach in total hip arthroplasty (SuperPATH)^4^. SuperPATH was invented based on surgical techniques from previous microposterior approaches, namely the supercapsular approach (SuperCap) developed by Stephen Murphy in 2004^5^ and the percutaneously assisted total hip approach (PATH) developed by Brad Penenberg in 2008^6^. The aim of the developers of SuperPATH was to maintain and combine the advantages of both microposterior approaches (Figs. 1 and 2). Numerous meta-analyses have already shown that SuperPATH significantly improves the short-term outcome of hip replacement compared to conventional approaches (CAs)^7–9^ and other minimally invasive hip approaches^10–13^. All of these meta-analyses^7–13^ concentrated on SuperPATH THA. Only two of them included some studies on SuperPATH HA^7,9^. The first English-language meta-analysis on SuperPATH concentrated on SuperPATH THA vs. CAs THA and performed a subgroup-analysis with a minor number of included studies on SuperPATH HA vs. CAs HA^7^. The meta-analysis by Ge et al.^9^ did not differentiate between SuperPATH THA and SuperPATH HA, which is a severe limitation. A recent scoping review including all publications on SuperPATH in PubMed showed that there is no meta-analysis in the literature examining the outcome of SuperPATH HA in patients with femoral neck fractures^14^. However, there is a fundamental difference between SuperPATH HA and SuperPATH THA. The additional stab incision for the acetabular cup positioning is not required in SuperPATH HA, as it was described by Bodrogi et al. in 2016^15^. In contrast, the CAs themselves do not differ depending on the choice between HA and THA. This difference might lead to even greater advantages of SuperPATH HA vs. CAs HA than SuperPATH THA vs. CAs THA already demonstrated.

The aim of the current study was to conduct a systematic review of literature and meta-analysis of randomized controlled trials comparing short-term outcomes of bipolar HA through SuperPATH and bipolar HA through CAs in patients with femoral neck fractures. The following PICO question was formulated: In human participants with femoral neck fractures, are the short-term outcomes of SuperPATH HA better than the short-term outcomes of CAs HA?

## Methods

After an initial literature search to check whether there were enough primary studies to conduct a meta-analysis, the study protocol was registered in PROSPERO on January 15, 2023 [CRD42023389353], available online at: https://www.crd.york.ac.uk/prospero/display_record.php?RecordID=389353. We followed the Preferred Reporting Items for Systematic reviews and Meta-Analyses (PRISMA) guidelines^16^. The PRISMA Checklist is provided in the supplement. We searched the following databases and checked citations of screened studies and reviews for relevant manuscripts until 25 August 2023: PubMed, CNKI, CENTRAL of The Cochrane Library, Clinical trials. Additional studies and gray literature were also searched in Google Scholar. The reference lists of found articles and similar meta-analyses were also checked for relevant studies. The literature search was carried out using the BOOLEAN search strategy, which was adapted to the syntax of the databases used: ((SuperPATH) OR (supercapsular percutaneously assisted total hip)).

### Study screening and selection

No restrictions on publication language and publication year were applied. The articles found were exported to a reference management software (Endnote Version × 9; Clarivate Analytics, London, UK). After removing duplicates, two reviewers (NR, PMK) independently selected articles by reviewing titles and abstracts. After viewing the full text of the selected articles, the two investigators independently decided whether to include them in the meta-analysis. In case of contradictory decisions, a consensus was reached after scientific discussion. The kappa coefficient was used to measure agreement between the two reviewers. Chinese articles have been translated using an artificial intelligence machine translator.

### Inclusion criteria

Types of studies:

randomized controlled trials (RCTs)

Types of participants:

human participants with a femoral neck fracture

Types of interventions:

bipolar HA through SuperPATH compared to bipolar HA through CAs

Types of outcome measures:

- Operation time (in min.): The operation time was defined as the time from skin incision to suture.
- Incision length (in cm): The incision length was defined as the length of the skin incision.
- Intraoperative blood loss (in mL): The intraoperative blood loss was defined as the volume of blood collected in the suction system.
- Postoperative drainage volume (in mL): The postoperative drainage volume was defined as the collected blood volume until the wound drainage was removed.
- Time to mobilization (in d): Time to mobilization was defined as the time interval from the end of surgery until the patient got out of bed and took his first steps with or without the assistance of physiotherapists or medical staff.
- Pain visual analog scale (VAS)^17,18^: Pain was measured with a subjective perception of the patient assessed using an objective measuring instrument. As in most cases, the VAS was used to determine pain intensity, with “10” being the most severe pain imaginable on a scale from 0 to 10.
- Harris Hip Score (HHS)^19^: The HHS, developed to evaluate the outcome of hip operations, collected points from assessments of four aspects of the hip condition: pain, function, degree of deformity, and range of motion. The higher the added score, the better the outcome, with “100” being the best result on a scale from 0 to 100.
- Hospitalization time (in d): The hospitalization time was defined as the time interval from admission to the hospital until the patient discharge.
- Complications: A surgical complication refers to an undesirable development or an unexpectedly difficult course from the time of surgery. The complications considered were as follows: dislocation, infection, periprosthetic fracture (intra- or postoperatively), deep vein thrombosis (with or without pulmonary artery embolism), hematoma, and reoperation or prosthesis revision. Depending on the individual RCTs, the postoperative complications were recorded in different follow-up periods, but in each case, these were short-term complications (≤ 1 year).

### Data extraction and analysis

Two reviewers (NR, PMK) independently extracted all relevant data: author’s name, publication year and origin of the RCTs, other RCT characteristics, study methods and quality, characteristics of the participants, details of the interventions, outcome parameters, and relevant additional information. The extracted data are available in the supplement. If relevant data were still missing after contacting the corresponding authors, the RCTs were excluded to ensure the high-quality inclusion of RCTs. If the RCTs provided different information from the intention-to-treat analysis (ITT) and the per-protocol analysis (PP), the numbers from the ITT analysis were used. The patient cohort characteristics of the SuperPATH group and the CAs group were summarized with unweighted descriptive measures and compared using the Mann–Whitney *U* test with a significance level α of 0.05.

### Quality assessment

We examined the selected RCTs for their quality. We performed a risk of bias and level of evidence assessment, using Cochrane’s Risk of Bias 2 (RoB 2) tool^20^, respectively according to the recommendations of the GRADE system^21^. Furthermore, we evaluated publication bias visually using funnel plots. In the funnel plot, the horizontal axis (“x-axis”) shows the mean difference (MD) in outcome between the two treatment groups for the individual RCTs, and the vertical axis (“y-axis”) shows the estimated standard error of the MD, i.e., the uncertainty of the estimated effect size. The vertical line is the overall effect estimated from the meta-analysis across all RCTs. The light gray triangle corresponds to the results based on the fixed effects model. The black triangle corresponds to the results based on the random effects model. Due to the small number of included studies, tests for funnel plots asymmetry were not performed^22^.

### Meta-analysis

The SuperPATH group was the experimental group and the CAs group was the control group. Both fixed effects and random effects models were calculated for each outcome. However, for the generalizability of the conclusions beyond the included studies, the interpretation focused on the results of the random effects model. For each RCT, MD was calculated as an unstandardized mean difference and its 95% confidence interval (CI) was calculated assuming unequal standard deviations in the two treatment groups. Across all RCTs, MD and its 95% CI was based on a random effects model. The study weighting was carried out using the inverse variance method. The between-study variance was estimated with the DerSimonian-Laird method. Heterogeneity was assessed using Cochrane’s Q-test (P-value < 0.10 indicates heterogeneity) and Higgins’ I^2^ test (low heterogeneity: < 25%, moderate heterogeneity: 25–75%, and high heterogeneity: > 75%)^23^. All statistical analyses were performed with netmeta and metafor packages in R software version 4.0.3.^24^. The results were analyzed based on the Cochrane manual for systematic reviews of interventions, Cochrane’s Review Manager version 5.3.

## Results

### Systematic Review

**Figure 3:**
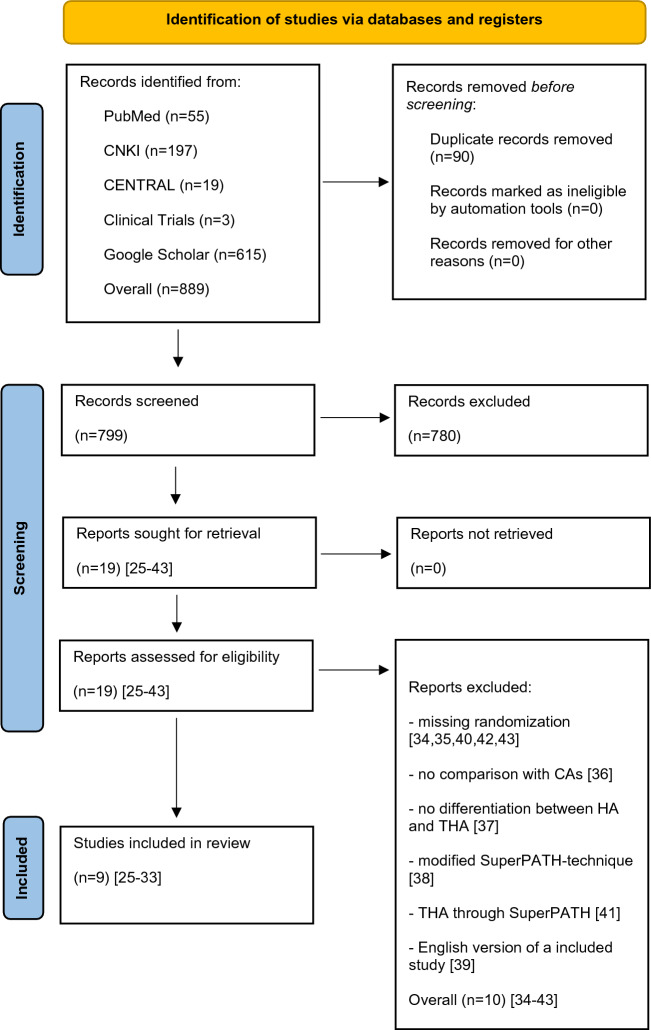
PRISMA flow chart of the literature search. *CNKI* China National Knowledge Infrastructure; *CENTRAL* Cochrane Central Register of Controlled Trials; *THA* total hip arthroplasty; *HA* hemiarthroplasty; *RCT* randomized controlled trial.

The initial literature search in the scientific medical databases yielded 799 unique records (Fig. 3). After reviewing the titles and abstracts, 19^25–43^ RCTs were selected by reviewers (κ = 1.0) for further consideration. Of these 19 RCTs, 10 RCTs^34–43^ were excluded after full-text screening (κ = 1.0) for the following reasons: (1) 5 RCTs^34,35,40,42,43^ were excluded due to lack of randomization, (2) one RCT compared two different SuperPATH groups with each other and not with a CAs^36^; (3) one RCT did not make any differentiation between HA ​​and THA^37^; (4) in one RCT a modified SuperPATH technique was used^38^; (5) one RCT compared SuperPATH THA to CAs HA^41^; (6) one RCT was an English-language version of the included study by Jia et al.^28^ with a partially identical patient cohort and with fewer outcome parameters^39^. Finally, a total of 9 RCTs^25–33^ were included in our meta-analysis.

### Characteristics of the included RCTs

The main features of the 9 included RCTs with overall 762 patients are shown in Table 1. The 9 RCTs were published between 2017 and 2021 in Chinese scientific journals. Five RCTs compared SuperPATH with a conventional posterolateral approach^25,29–31,33^, 2 RCTs compared SuperPATH with a conventional posterior approach^26,28^, 2 RCTs compared SuperPATH with a conventional lateral approach^27,32^. Of the included patients, 377 were operated through SuperPATH, and 385 were patients operated through CAs. Of these 385 patients, 220 were operated on through posterolateral CA, 82 through posterior CA, and 83 through lateral CA. The RCTs examined the short-term outcome (≤ 1 year) with a mean follow-up time of 7.6 months (range: 6–10).

**Table 1:** Features of the included RCTs.

Author	Publication year	Origin	Language	English Abstract	Approach	Patients, N	Follow-up period, months	Outcome parameters
Dai GH et al.^25^	2019	China	Chinese	Yes	S	61	6	1, 2, 3, 4, 5, 6, 7, 8, 9, 10, 11, 12
					PL	67	6	
Ding B et al.^26^	2018	China	Chinese	No	S	50	–	1, 2, 3, 6
					P	50	–	
Huang J et al.^27^	2021	China	Chinese	Yes	S	35	–	1, 2, 3, 5, 6, 7, 11, 12
					L	35	–	
Jia J et al.^28^	2017	China	Chinese	Yes	S	32	8.3	1, 2, 3, 4, 5, 8, 10, 12
					P	32	8.3	
Wang X and Tian J^29^	2021	China	Chinese	No	S	50	–	1, 2, 3, 4, 5, 9, 11
					PL	50	–	
Xia LZ et al.^30^	2018	China	Chinese	Yes	S	30	10	1, 2, 3, 5, 6, 7, 8, 9, 10, 12
					PL	32	10	
Xu G et al.^31^	2018	China	Chinese	Yes	S	46	–	1, 2, 3, 4, 5, 10, 11, 12
					PL	46	–	
Zhao S^32^	2021	China	Chinese	No	S	48	–	1, 2, 3, 5, 6, 7, 11
					L	48	–	
Zhao L et al.^33^	2019	China	Chinese	No	S	25	6	1, 2, 8, 10, 11, 12
					PL	25	6	

L, lateral; P, posterior; PL, posterolateral; S, SuperPATH; 1, operation time; 2, incision length; 3, intraoperative blood loss; 4, postoperative drainage volume; 5, time to mobilization; 6, pain VAS ≤ 1 week postoperatively; 7, pain VAS 1–3 months postoperatively; 8, HHS ≤ 1 week postoperatively; 9, HHS 3 months postoperatively; 10, HHS 6 months postoperatively; 11, hospitalization time; 12, complications.

### Characteristics of the patient cohort

All included patients had a unilateral femoral neck fracture, mostly dislocated (98.9%). The mean age of the patient cohort was 74.9 years (range: 67.1–82.8) and 47.2% of the patients were men. Only 2 studies^25,32^ reported the Body Mass Index (BMI), which averaged 24 kg/m^2^. The mean preoperative HHS of the patient cohort was 26.2 points. Few studies reported information on the comorbidities. The most frequent comorbidities were arterial hypertension with 55.1%, coronary artery disease (CAD) with 22.5%, and diabetes mellitus with 21.9%. These relevant patient characteristics did not differ significantly between the SuperPATH and the CAs group (Table 2). Further details on patient characteristics are given in Table 3.

**Table 2:** Comparison of the patient characteristics between the SuperPATH group and the CAs group using the Mann–Whitney *U* Test with a significance level α of 0,05.

	Total	SuperPATH	CAs	p-value
N; mean/median (1st interquartile—3rd interquartile)				
Age	18; 74.9/73.7 (70.4–80.8)	9; 74.8/73.2 (70.4–81.0)	9; 75.1/74.1 (70.5–80.7)	0.999
Male	18; 47.2/46.0 (37.3–58.3)	9; 46.7/48.0 (40.6–54.3)	9; 47.7/42.0 (37.3–60.4)	0.911
BMI	4; 24.0/23.9 (22.6–25.4)	2; 24.1/24.1 (22.6–25.6)	2; 23.8/23.8 (22.5–25.1)	0.667
Hypertension	8; 51.1/43.5 (40.2–60.9)	4; 51.7/49.3 (42.5–60.9)	4; 50.6/41.1 (39.9–61.3)	0.686
CAD	6; 22.5/24.3 (18.8–26.9)	3; 21.8/24.6 (12.5–28.3)	3; 23.2/23.9 (18.8v26.9)	0.999
Diabetes	8; 21.9/21.8 (17.8–27.4)	4; 22.1/23.7 (16.3–28.0)	4; 21.6/21.8 (18.9–24.4)	0.886
COPD	4; 4.5/4.5 (3.5–5.5)	2; 4.5/4.5 (4.0–5.0)	2; 4.5/4.5 (3.0–6.0)	0.999
HHS preop	8; 26.2/24.1 (14.6–37.7)	4; 26.4/24.3 (14.6–38.2)	4; 26.0/24.1 (14.6–37.4)	0.886

CAs, conventional approaches; BMI, Body Mass Index; CAD, coronary artery disease; COPD, chronic obstructive pulmonary disease; HHS preop., Harris Hip Score preoperatively.

**Table 3:** Patient characteristics.

Author	Patients with femoral neck fractures, N	Dislocated femoral neck fractures, N (%)	HHS preoperatively, points, SD	Age, years, SD	Range of the age, years, min. –max.	Male gender, N (%)	BMI, kg/m^2^	Hypertension, N (%)	CAD, N (%)	Diabetes, N (%)	COPD, N (%)
Dai GH et al.^25^	61	61 (100)	33.2 ± 1.7	69.9	65–80	22 (36.1)	25.63	24 (39.3)	15 (24.6)	17 (27.9)	4 (6.6)
	67	67 (100)	32.6 ± 1.8	70.3	65–79	25 (37.3)	25.13	26 (38.8)	18 (26.9)	18 (26.9)	6 (9)
Ding B et al.^26^	50	–	–	81.2 ± 5.9	–	22 (44)	–	–	–	–	–
	50	–	–	80.8 ± 5.6	–	21 (42)	–	–	–	–	–
Huang J et al.^27^	35	35 (100)	–	73.2 ± 4.2	67–82	21 (60)	–	–	–	–	–
	35	35 (100)	–	74.1 ± 4.2	66–84	23 (65.7)	–	–	–	–	–
Jia J et al.^28^	32	32 (100)	15.4 ± 2.8	77.1 ± 2.3	–	13 (40.6)	–	22 (68.8)	4 (12.5)	9 (28.1)	5 (15.6)
	32	32 (100)	15.6 ± 2.4	78.5 ± 2.6	–	11 (34.4)	–	26 (81.3)	6 (18.8)	7 (21.9)	3 (9.4)
Wang X and Tian J^29^	50	50 (100)	43.2 ± 8.7	67.8 ± 7.4	62–78	26 (52)	–	–	–	–	–
	50	50 (100)	42.2 ± 8.9	67.2 ± 6.6	61–76	27 (54)	–	–	–	–	–
Xia LZ et al.^30^	30	25 (83)	–	81 ± 4.6	74–91	8 (26.7)	–	16 (53)	–	4 (13)	–
	32	30 (94)	–	80.7 ± 4,3	67–89	11 (34.4)	–	13 (41)	–	5 (16)	–
Xu G et al.^31^	46	46 (100)	13.7	70.8 ± 6.1	61–79	25 (54.3)	–	21 (45.7)	13 (28.3)	9 (19.6)	–
	46	46 (100)	13.5	71 ± 6	60–82	28 (60.9)	–	19 (41.3)	11 (23.9)	10 (21,7)	–
Zhao S^32^	48	48 (100)	–	70.4 ± 1.5	–	28 (58.3)	22.6 ± 1.5	–	–	–	–
	48	48 (100)	–	70.5 ± 1.5	–	29 (60.4)	22.5 ± 1.5	–	–	–	–
Zhao L et al.^33^	25	–	–	81.5 ± 5.2	–	12 (48)	–	–	–	–	–
	25	–	–	82.8 ± 6.3	–	10 (40)	–	–	–	–	–

HHS, Harris Hip Score; SD, standard deviation; BMI, Body Mass Index; CAD, coronary artery disease; COPD, chronic obstructive pulmonary disease.

### Quality assessment

**Figure 4:**
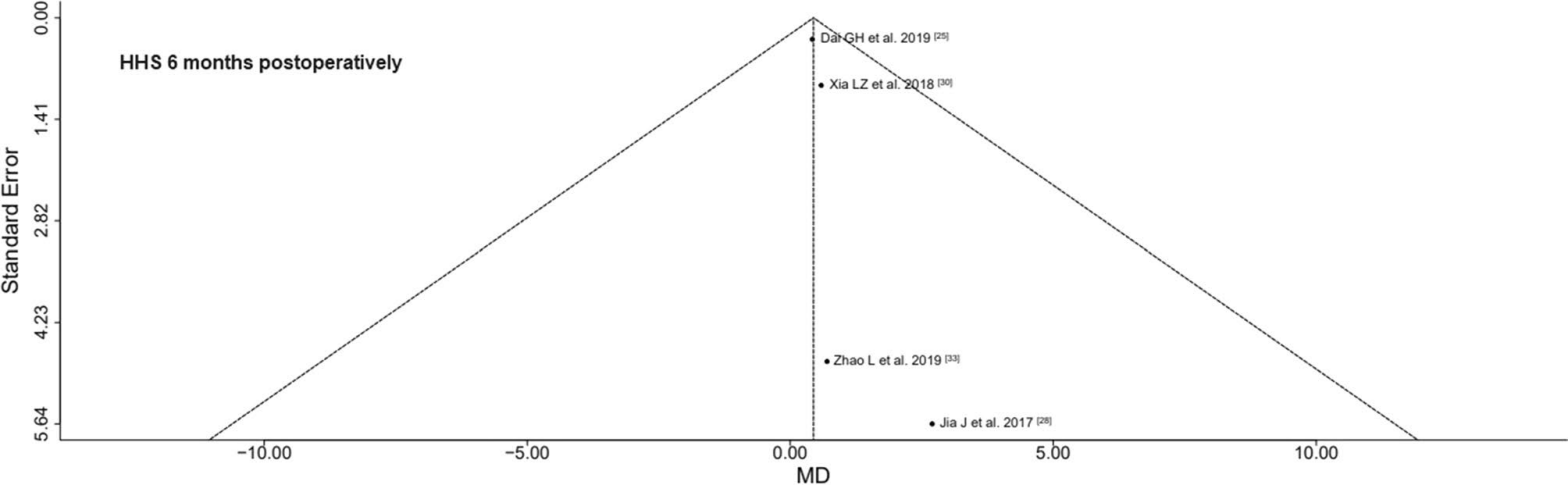
Funnel plot of the HHS 6 months postoperatively. X-axis shows the MD in outcome between the two treatment groups, y-axis shows the estimated standard error of the MD. Vertical line is the overall effect estimated from the RCT meta-analysis. Black triangle corresponds to the the random effects model results. *MD* mean difference; *RCT* randomized controlled trial.

All 9 RCTs were rated with a moderate risk of bias (Table 4). The quality of evidence of the outcome parameters was rated as follows: the HHS 6 months postoperatively had a moderate quality of evidence, the intraoperative blood loss and the pain VAS 1–3 months postoperatively had a low quality of evidence; all other outcome parameters had a very low quality of evidence (Table 5). Overall, the funnel plots were broadly symmetrical, possibly indicating low to high publication bias. The pain VAS ≤ 1 week postoperatively, the pain VAS 1–3 months postoperatively, the HHS 3 months postoperatively, the HHS 6 months postoperatively, and the hospitalization time were rated with a low publication bias. The operation time, the intraoperative blood loss, the postoperative drainage volume, the time to mobilization, and the HHS ≤ 1 week postoperatively were rated with a moderate publication bias. The incision length was rated with a high publication bias. A representative funnel plot of the HHS 6 months postoperatively is shown in Fig. 4. The funnel plots of all 11 outcome parameters analyzed are presented in the supplement. However, it is unlikely that the short-term outcome of SuperPATH HA is not superior to CAs HA, because a quick look at several non-RCTs in PubMed, CNKI, CENTRAL of The Cochrane Library and Clinical Trials did not show any difference from our results.

**Table 4:** Risk of bias assessment.

Study	Random sequence generation	Allocation concealment	Blinding	Complete outcome data	Selective reporting	Other sources of bias	Overall risk of bias
Dai GH et al.^25^	** + **	** + **	*?*	** + **	** + **	** + **	*Moderate RoB*
Ding B et al.^26^	** + **	** + **	*?*	*?*	** + **	** + **	*Moderate RoB*
Huang J et al.^27^	** + **	** + **	*?*	*?*	** + **	** + **	*Moderate RoB*
Jia J et al.^28^	** + **	** + **	*?*	** + **	** + **	** + **	*Moderate RoB*
Wang X and Tian J^29^	** + **	** + **	*?*	*?*	** + **	** + **	*Moderate RoB*
Xia LZ et al.^30^	** + **	** + **	*?*	** + **	** + **	** + **	*Moderate RoB*
Xu G et al.^31^	** + **	** + **	*?*	*?*	** + **	** + **	*Moderate RoB*
Zhao S^32^	** + **	** + **	*?*	*?*	** + **	** + **	*Moderate RoB*
Zhao L et al.^33^	** + **	** + **	*?*	*?*	** + **	** + **	*Moderate RoB*

(+): low RoB; (?): unclear, some concerns, or moderate RoB; (–): high RoB. RoB: risk of bias.

**Table 5:** Level of evidence assessment according to GRADE recommendations.

No. of studies	Design	Risk of bias	Inconsistency	Indirectness	Imprecision	Other considerations	Quality of evidence
Operation time							
9	RCT	Moderate	Serious	No serious indirectness	No serious imprecision	All studies were from China	Low
Incision length							
9	RCT	Moderate	Serious	No serious indirectness	No serious imprecision	All studies were from China	Low
Intraoperative blood loss							
8	RCT	Moderate	Serious	No serious indirectness	Serious	All studies were from China	Very low
Postoperative drainage volume							
4	RCT	Moderate	Serious	No serious indirectness	No serious imprecision	All studies were from China	Low
Time to mobilization							
7	RCT	Moderate	Serious	No serious indirectness	No serious imprecision	All studies were from China	Low
Pain VAS ≤ 1 week postoperatively							
5	RCT	Moderate	Serious	No serious indirectness	No serious imprecision	All studies were from China	Low
Pain VAS 1–3 months postoperatively							
4	RCT	Moderate	Serious	No serious indirectness	Serious	All studies were from China	Very low
HHS ≤ 1 week postoperatively							
4	RCT	Moderate	Serious	No serious indirectness	No serious imprecision	All studies were from China	Low
HHS 3 months postoperatively							
3	RCT	Moderate	Serious	No serious indirectness	No serious imprecision	All studies were from China	Low
HHS 6 months postoperatively							
4	RCT	Moderate	No serious inconcistency	No serious indirectness	No serious imprecision	All studies were from China	Moderate
Hospitalization time							
6	RCT	Moderate	Serious	No serious indirectness	No serious imprecision	All studies were from China	Low

RCT, randomized controlled trial; HHS, Harris Hip Score; VAS, Visual Analog Scale.

### Meta-analysis

#### Operation time

**Figure 5:**
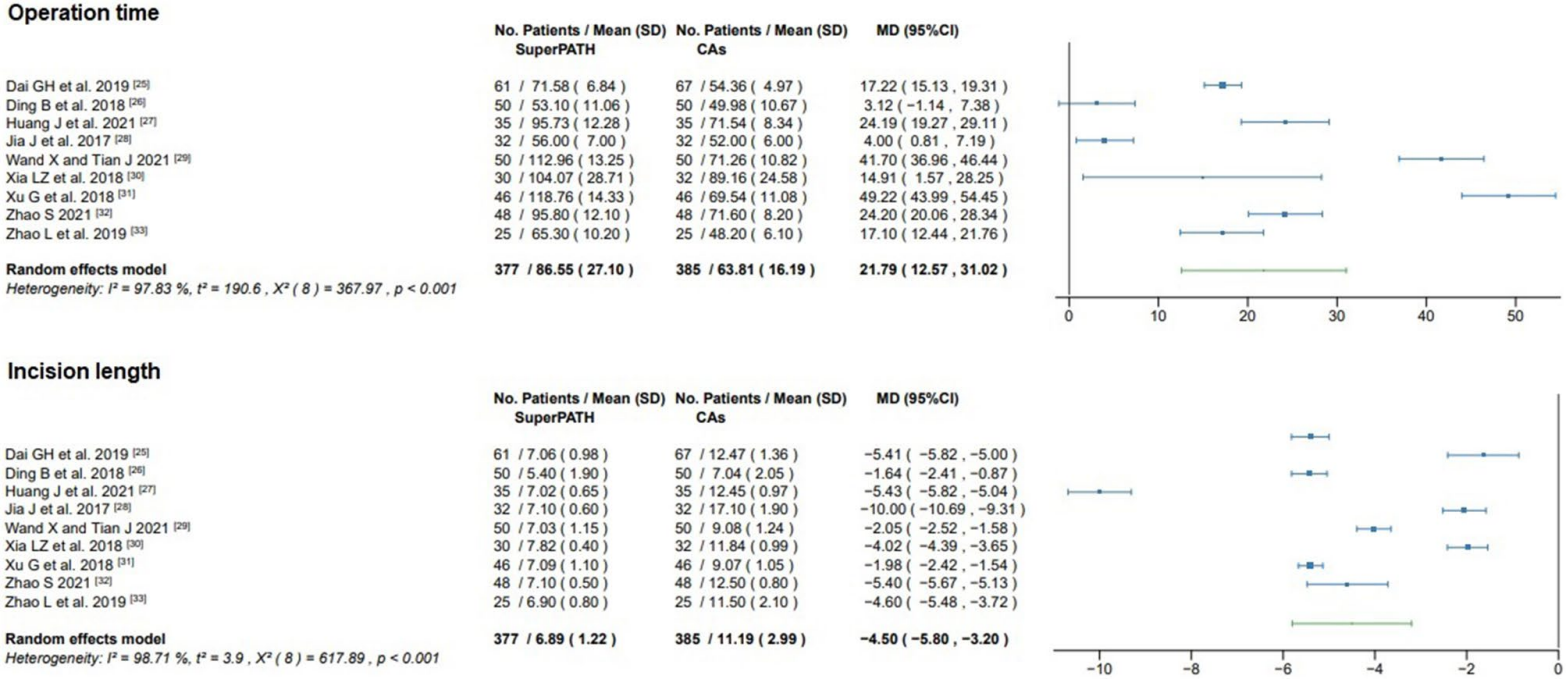
Forest plot of the operation time (in min.) and incision length (in cm). *SD* standard deviation; *CAs* conventional approaches; *MD* mean difference; *CI* confidence interval.

Data on the operation time of 762 patients were pooled from 9 RCTs (I^2^ = 98%, p < 0.01, Fig. 5). The SuperPATH group consisted of 377 patients with a weighted mean operation time of 86.6 min. The CAs group consisted of 385 patients with a weighted mean operation time of 63.8 min. The operation time of the SuperPATH group was 21.8 min. significantly longer than the operation time of the CAs group (MD = 21.79, 95% CI 12.57 to 31.02).

#### Incision length

Data on the incision length of 762 patients were pooled from 9 RCTs (I^2^ = 99%, p < 0.01, Fig. 5). The SuperPATH group consisted of 377 patients with a weighted mean incision length of 6.9 cm. The CAs group consisted of 385 patients with a weighted mean incision length of 11.2 cm. The incision length of the SuperPATH group was 4.5 cm significantly shorter than the incision length of the CAs group (MD = − 4.50; 95% CI − 5.80 to − 3.20).

#### Intraoperative blood loss

**Figure 6:**
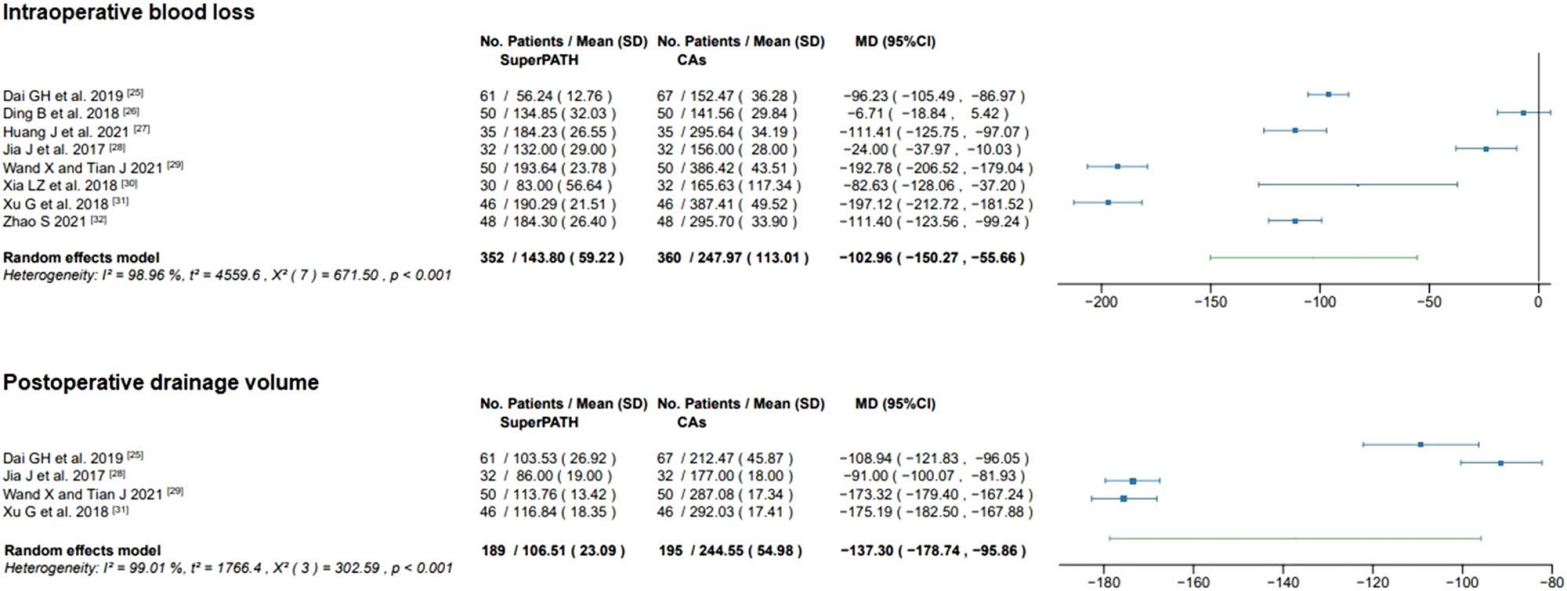
Forest plot of the intraoperative blood loss (in ml) and postoperative drainage volume (in ml). *SD* standard deviation; *CAs* conventional approaches; *MD* mean difference; *CI* confidence interval.

Data on the intraoperative blood loss of 712 patients were pooled from 8 RCTs (I^2^ = 99%, p < 0.01, Fig. 6). The SuperPATH group consisted of 352 patients with a weighted mean intraoperative blood loss of 143.8 mL. The CAs group consisted of 360 patients with a weighted mean intraoperative blood loss of 248.0 mL. The intraoperative blood loss of the SuperPATH group was 103.0 mL significantly less than the intraoperative blood loss of the CAs group (MD = − 102.96, 95% CI − 150.27 to − 55.66).

#### Postoperative drainage volume

Data on the postoperative drainage volume of 384 patients were pooled from 4 RCTs (I^2^ = 99%, p < 0.01, Fig. 6). The SuperPATH group consisted of 189 patients with a weighted mean postoperative drainage volume of 106.5 mL. The CAs group consisted of 195 patients with a weighted mean postoperative drainage volume of 244.6 mL. The postoperative drainage volume of the SuperPATH group was 137.3 mL significantly less than the postoperative drainage volume of the CAs group (MD = − 137.30, 95% CI − 178.74 to − 95.86).

#### Pain VAS ≤ 1 week postoperatively

**Figure 7:**
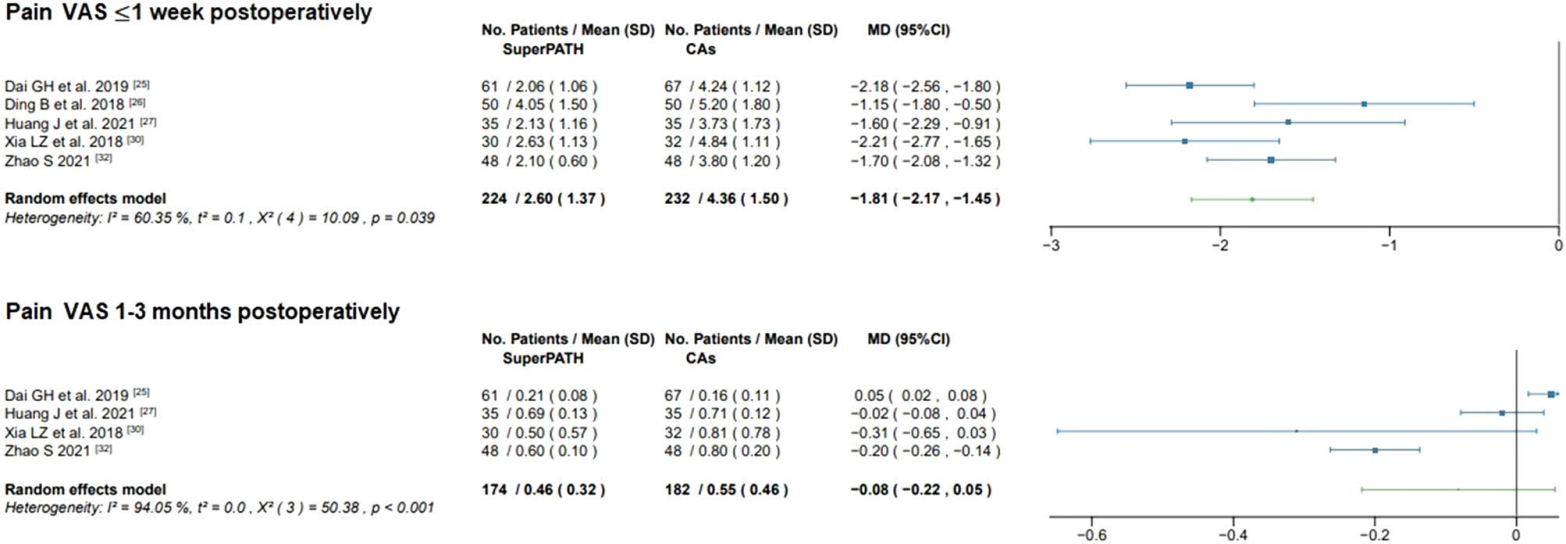
Forest plot of the pain VAS ≤ 1 week and 1–3 months postoperatively (in points). *SD* standard deviation; *CAs* conventional approaches; *MD* mean difference; *CI* confidence interval.

Data on the pain VAS ≤ 1 week postoperatively of 456 patients were pooled from 5 RCTs (I^2^ = 60%, p = 0.04, Fig. 7). The SuperPATH group consisted of 224 patients with a weighted mean pain VAS ≤ 1 week postoperatively of 2.6 points. The CAs group consisted of 232 patients with a weighted mean pain VAS ≤ 1 week postoperatively of 4.4 points. The pain VAS ≤ 1 week postoperatively of the SuperPATH group was 1.8 points significantly lower than the pain VAS ≤ 1 week postoperatively of the CAs group (MD = − 1.81; 95% CI − 2.17 to − 1.45).

#### Pain VAS 1–3 months postoperatively

Data on the pain VAS 1–3 months postoperatively of 356 patients were pooled from 4 RCTs (I^2^ = 94%, p < 0.01, Fig. 7). The SuperPATH group consisted of 174 patients with a weighted mean pain VAS 1–3 months postoperatively of 0.5 points. The CAs group consisted of 182 patients with a weighted mean pain VAS 1–3 months postoperatively of 0.6 points. There was no statistically significant difference in pain VAS 1–3 months postoperatively between the SuperPATH group and the CAs group (MD = − 0.08; 95% CI − 0.22 to 0.05).

#### HHS ≤ 1 week postoperatively

**Figure 8:**
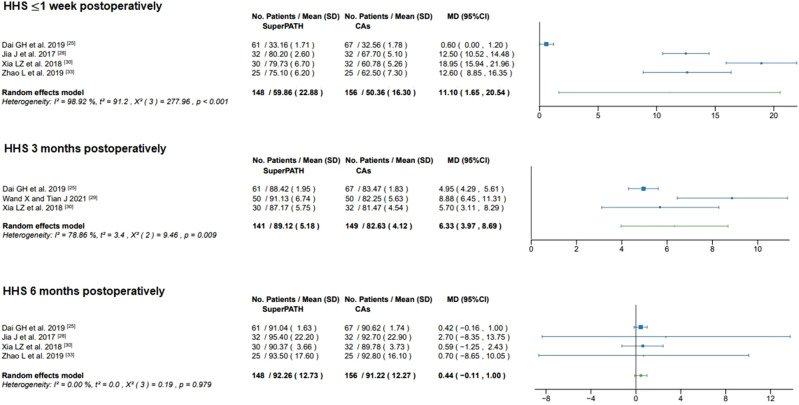
Forest plot of the HHS ≤ 1 week, 3 months and 6 months postoperatively (in points). *SD* standard deviation; *CAs* conventional approaches; *MD* mean difference; *CI* confidence interval.

Data of 304 patients were pooled from 4 RCTs (I^2^ = 99%, p < 0.01, Fig. 8). The SuperPATH group consisted of 148 patients with a weighted mean HHS ≤ 1 week postoperatively of 59.9 points. The CAs group consisted of 156 patients with a weighted mean HHS ≤ 1 week postoperatively of 50.4 points. The HHS ≤ 1 week postoperatively of the SuperPATH group was 11.1 points significantly higher than the HHS ≤ 1 week postoperatively of the CAs group (MD = 11.10; 95% CI 1.65 to 20.54).

#### HHS 3 months postoperatively

Data of 290 patients were pooled from 3 RCTs (I^2^ = 79%, p = 0.01, Fig. 8). The SuperPATH group consisted of 141 patients with a weighted mean HHS 3 months postoperatively of 89.1 points. The CAs group consisted of 149 patients with a weighted mean HHS 3 months postoperatively of 82.6 points. The HHS 3 months postoperatively of the SuperPATH group was 6.3 points significantly higher than the HHS 3 months postoperatively of the CAs group (MD = 6.33; 95% CI 3.97 to 8.69).

#### HHS 6 months postoperatively

Data of 304 patients were pooled from 4 RCTs (I^2^ = 0%, p = 0.98, Fig. 8). The SuperPATH group consisted of 148 patients with a weighted mean HHS 6 months postoperatively of 92.3 points. The CAs group consisted of 156 patients with a weighted mean HHS 6 months postoperatively of 91.2 points. There was no statistically significant difference in HHS 6 months postoperatively between the SuperPATH group and the CAs group (MD = 0.44; 95% CI − 0.11 to 1.00).

#### Time to mobilization

**Figure 9:**
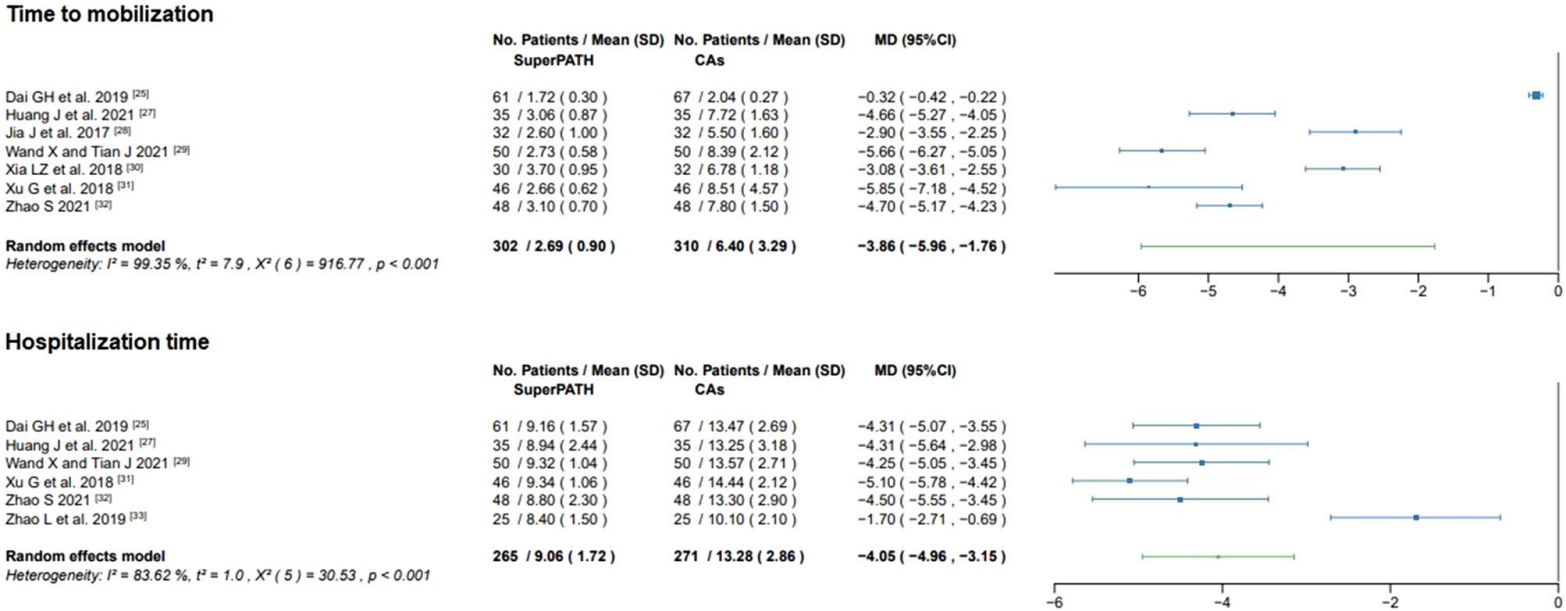
Forest plot of the time to mobilization (in d) and hospitalization time (in d). SD: standard deviation; *CAs* conventional approaches; *MD* mean difference; *CI* confidence interval.

Data on the time to mobilization of 612 patients were pooled from 7 RCTs (I^2^ = 99%, p < 0.01, Fig. 9). The SuperPATH group consisted of 302 patients with a weighted mean time to mobilization of 2.7 days. The CAs group consisted of 310 patients with a weighted mean time to mobilization of 6.4 days. The time to mobilization of patients in the SuperPATH group was 3.9 days significantly shorter than the time to mobilization of patients in the CAs group (MD = − 3.86; 95% CI − 5.96 to − 1.76).

#### Hospitalization time

Data of 536 patients were pooled from 6 RCTs (I^2^ = 84%, p < 0.01, Fig. 9). The SuperPATH group consisted of 265 patients with a weighted mean hospitalization time of 9.1 days. The CAs group consisted of 271 patients with a weighted mean hospitalization time of 13.3 days. The hospitalization time of the SuperPATH group was 4.1 days significantly shorter than the hospitalization time of the CAs group (MD = − 4.05; 95% CI − 4.96 to − 3.15).

#### Complications

The outcome parameter “complications” was presented descriptively (Table 6), since the extracted data from the 9 included RCTs^25–33^ was insufficient to conduct a meta-analysis. Information on the complication outcomes was reported in six^24,27,28,30,31,34^ of 9 RCTs (Table 6). Three^25,28,30^ of these 6 RCTs did not detect complications either in the SuperPATH group or in the CAs group. The RCT by Huang et al.^27^ reported one dislocation in the CAs group compared to none in the SuperPATH group. Furthermore, 4 cases of postoperative wound infections were reported in the CAs group compared to 1 case in the SuperPATH group^27^. The RCT by Xu et al.^31^ reported one dislocation in the CAs group compared to none in the SuperPATH group. Furthermore, 5 cases of postoperative wound infections were reported in the CAs group compared to 1 case in the SuperPATH group^31^. The RCT by Zhao et al.^33^ reported 2 cases of deep vein thrombosis in the CAs group compared to none in the SuperPATH group.

**Table 6:** Number of patients with complications.

Author/RCT	Approach	Overall complications	Dislocation	Infection	Periprosthetic frakture	Deep vein thrombosis	Haematoma	Reoperation/Revision
Dai GH et al.^25^	SuperPATH	0	0	0	0	0	0	0
	CAs	0	0	0	0	0	0	0
Ding B et al.^26^	SuperPATH	–	–	–	–	–	–	–
	CAs	–	–	–	–	–	–	–
Huang J et al.^27^	SuperPATH	1	0	1	0	0	0	0
	CAs	5	1	4	0	0	0	0
Jia J et al.^28^	SuperPATH	0	0	0	0	0	0	0
	CAs	0	0	0	0	0	0	0
Wang X and Tian J^29^	SuperPATH	–	–	–	–	–	–	–
	CAs	–	–	–	–	–	–	–
Xia LZ et al.^30^	SuperPATH	0	0	0	0	0	0	0
	CAs	0	0	0	0	0	0	0
Xu G et al.^31^	SuperPATH	1	0	1	0	0	0	0
	CAs	6	1	5	0	0	0	0
Zhao S^32^	SuperPATH	–	–	–	–	–	–	–
	CAs	–	–	–	–	–	–	–
Zhao L et al.^33^	SuperPATH	0	0	0	0	0	0	0
	CAs	2	0	0	0	2	0	0

RCT, randomized controlled trials; CAs, conventional approaches.

## Discussion

A recent scoping review of all publications on SuperPATH in PubMed attempted to determine the nature, extent and quality of the current research evidence on SuperPATH and identify areas for further investigation^14^. In this context, the present meta-analysis aimed to close the existing gap^14^ in the specialist literature on SuperPATH. To the best of our knowledge, this is the first meta-analysis of patients with femoral neck fractures comparing SuperPATH HA to CAs HA, with the exception of another meta-analysis that concentrated on SuperPATH THA and evaluated SuperPATH HA in a minor subgroup analysis^7^. The further value of this work is the inclusion of RCTs and the employment of high-quality statistical methods. This study is a part of a series of publications of the first author, whose main research area is hip arthroplasty and especially the novel SuperPATH approach^7,8,10–13^.

Nine RCTs^25–33^ with 762 patients were included in this meta-analysis. Of the included patients, 377 were operated through SuperPATH, and 385 patients were operated through CAs. All 9 RCTs had a moderate risk of bias. The HHS 6 months postoperatively had a moderate quality of evidence, the intraoperative blood loss and the pain VAS 1–3 months postoperatively had a low quality of evidence, and all other outcome parameters had a very low quality of evidence. Five out of 11 outcome parameters had a low publication bias. Five out of 11 outcome parameters had a moderate publication bias. One out of 11 outcome parameters had a high publication bias.

In general, our meta-analysis indicated that SuperPATH HA was superior to CAs HA regarding the investigated outcomes. SuperPATH HA showed better results on decreasing incision length, intraoperative blood loss, postoperative drainage volume, time to mobilization, early postoperative pain intensity, and hospitalization time. Moreover, SuperPATH HA improved early postoperative functional outcome. However, SuperPATH HA and CAs HA had comparable later short-term postoperative pain intensity and functional outcome.

As there exists no previous comparable study on this topic, we discussed the outcome parameters examined point by point comparing them to similar meta-analyses^7–9,44–46^. The first English-language meta-analysis of SuperPATH by Ramadanov et al.^7^ was published in 2020 and compared SuperPATH THA with CAs THA with a minor subgroup analysis of SuperPATH HA vs. CAs HA. The update of this meta-analysis, called Meta-SuCAs-2, compared only SuperPATH THA with CAs THA^8^. Another 2021 meta-analysis by Ge et al.^9^ did not differentiate between SuperPATH HA and SuperPATH THA, which is a severe limitation. Furthermore, there are three meta-analyses of direct anterior approach (DAA) HA vs. CAs HA^44–46^. The comparison between SuperPATH HA and DAA HA is interesting because DAA has long been considered the leading approach in short-term THA outcomes. This appears to be changing in favor of SuperPATH since its introduction one decade ago^10–12^.

The operation time was the only outcome parameter in which SuperPATH HA showed worse results than CAs HA. SuperPATH HA had a 21.8 min. significantly longer operation time than CAs HA. This supposed disadvantage of SuperPATH HA must be put into perspective. Too long operation times are associated with higher complication rates^47,48^. In contrast, there is no evidence that noticeably short operation times would lead to a reduction in complication rates. Rather, an average, moderate operation time must be assumed, which is associated with low complication rates. This has not yet been determined for HA in patients with femoral neck fractures. In THA, this low-risk operation time is about 80 min, which is shown in a 2019 analysis of 89,802 cases by Surace et al.^48^. Compared to this recommendation, the weighted mean operation time of SuperPATH HA was 86.6 min. and the weighted mean operation time of CAs was 63.8 min. A recent scoping review^14^ of the current SuperPATH literature concluded, based on the studies reviewed, that there is a clear learning curve for the SuperPATH technique. Using operation time as a surrogate, the learning curve begins to rise after 40–50 SuperPATH cases. This means that the operation time of SuperPATH can be further reduced as the surgeon becomes more familiar with the technique. The three meta-analyses of DAA HA found no significant differences in operation time compared to CAs HA^44–46^. The three meta-analyses of SuperPATH vs. CAs showed inconsistent results^7–9^. While Ge et al.^9^ found no significant differences in operation time, the other two meta-analyses showed that SuperPATH had a 14.3–18.4 min. longer operation time compared to CAs^7,8^.

On the one hand, the incision length has cosmetic relevance, on the other hand, it reflects the intraoperative tissue traumatization to a certain extent. The lower tissue traumatization of the small skin incision relates mainly to the superficial tissue. In some cases, however, there are surgical techniques that can cause more deep tissue damage, even using smaller skin incisions. The weighted mean incision length of SuperPATH HA was 6.9 cm, ranging from 5.4–7.8 cm. It was 4.5 cm significantly shorter than the incision length of CAs HA. The three meta-analyses of DAA HA vs. CAs HA did not examine the incision length^44–46^. The other three meta-analyses found a smaller incision length through SuperPATH compared to CAs^7–9^. In Meta-SuCAs-2^8^, the mean incision length of SuperPATH THA was 7 cm, ranging from 5.8 to 10.4 cm. When comparing, it is noticeable that the ranges of the incision length of SuperPATH HA are somewhat shorter than the ranges of the incision length of SuperPATH THA. This can be explained by the additional stab incision that is required in SuperPATH THA^4^ and that can be omitted in SuperPATH HA^15^. However, in DAA hip replacement it cannot be assumed that the incision length differs due to the choice between THA and HA. The incision length of DAA THA has already been determined and it was significantly longer than the incision length of SuperPATH THA^10–12^. Thus, SuperPATH meets one of the well-known conditions to be considered a minimally invasive approach better than DAA, namely having an incision length ≤ 10 cm in hip replacement.

The blood loss reflects the intraoperative trauma. It was measured intraoperatively and postoperatively. The weighted mean intraoperative blood loss of SuperPATH HA was 143.8 mL, ranging from 56.2 to 193.6 mL. SuperPATH HA had a 103.0 mL significantly lower intraoperative blood loss compared to CAs HA. The weighted postoperative drainage volume of SuperPATH HA was 106.5 mL, ranging from 86 to 116.8 mL. SuperPATH HA had a 137.3 mL significantly lower postoperative drainage volume compared to CAs HA. Any blood loss requiring transfusion should be considered clinically relevant, although there is no evidence in the literature as to what specific blood loss constitutes a minimal clinically important difference (MCID). The difference in intraoperative and postoperative blood loss between SuperPATH HA and CAs HA totaling approximately 240 mL is not irrelevant. Such a difference can certainly prevent a blood transfusion in some cases. The two meta-analyses by Kunkel et al.^44^ and by Khan et al.^45^ showed no significant difference in perioperative blood loss between DAA HA and CAs HA. In Meta-SuCAs-2^8^, SuperPATH THA had a mean intraoperative blood loss of 132 mL, ranging from 89 to 1108 mL. The intraoperative blood loss was only 61 mL lower compared to CAs THA^8^. The reduced intraoperative blood loss of SuperPATH HA compared to SuperPATH THA can certainly be explained by the reaming of the acetabular cup in THA. The additional stab incision required for THA may also make a small contribution to the overall blood loss.

The weighted mean time to mobilization of SuperPATH HA was 2.7 days, ranging from 1.7 to 3.7 days. SuperPATH HA had a 3.9 days significantly shorter time to mobilization compared to CAs. The specialist literature is sparse with reliable studies on the time to mobilization after HA according to different hip approaches. Nevertheless, the outcome parameter is important, since early mobilization is necessary, particularly in older patients, in order to prevent concomitant diseases (e.g. pneumonia, decubitus).

Pain is a subjective perception of the patient, which was recorded with the objective measurement instrument pain VAS. The pain intensity was measured ≤ 1 week and 1–3 months postoperatively. The recording of the pain intensity is also part of the determination of the HHS. The pain intensity has a clear effect on the patient's comfort. Even more important is that the high intensity of pain can slow down the patient's time to mobilization with all the possible complications that may result from this delay. The weighted pain VAS ≤ 1 week postoperatively of SuperPATH HA was 2.6 points, ranging from 2.1 to 4.1 points. It was 1.8 points significantly lower than the pain VAS ≤ 1 week postoperatively of CAs HA. A recent comparative study by Danoff et al.^49^ found that a pain difference of 18.6 mm for THA patients, measured on a 100 VAS-P scale, is considered to be a MCID. This would correspond to a difference of 1.9 points, applied to the 10-point pain VAS. This difference was almost achieved by SuperPATH HA compared to CAs HA. The results for the pain VAS 1–3 months postoperatively between SuperPATH HA vs. CAs HA were not significantly different, emphasizing that the strength of SuperPATH HA is to improve the early short-term HA outcome. Other meta-analyses came to the same conclusion. In the first English-language meta-analysis of SuperPATH, the pain VAS 1 day postoperatively of SuperPATH was 0.8 points lower and the pain VAS 7 days postoperatively of SuperPATH was 1.4 points lower in comparison to CAs^7^. Later, it became comparable at 3 and 12 months postoperatively. In the meta-analysis by Ge et al.^9^, the pain VAS 7 days postoperatively of SuperPATH was 1.3 points lower compared to CAs. Again, it became comparable at 1, 3, 6, and 12 months postoperatively. In Meta-SuCAs-2^8^, the pain VAS 1 day postoperatively of SuperPATH THA was 1 point lower and the pain VAS 3 days postoperatively of SuperPATH THA was 1.2 points lower compared to CAs THA. Accordingly, SuperPATH HA seems to deliver even better pain relief results than SuperPATH THA. Literature is contradictive on the results of DAA HA in postoperative pain scores compared to CAs HA^44,45^.

The HHS is one of the most common scores that can be used to assess hip function after surgery. In the literature on hip arthroplasty, the hip function measured using HHS or other measurement tools is usually regarded as the most important outcome parameter. The weighted mean HHS ≤ 1 week postoperatively of SuperPATH HA was 59.9 points, ranging from 33.2 to 80.2 points. SuperPATH HA had a 11.1 points significantly higher HHS ≤ 1 week postoperatively compared to CAs HA. The weighted mean HHS 3 months postoperatively of SuperPATH HA was 89.1 points, ranging from 87.2 to 91.1 points. SuperPATH HA had a 6.3 points significantly higher HHS 3 months postoperatively compared to CAs HA. The weighted mean HHS 6 months postoperatively of SuperPATH HA was 92.3 points, ranging from 90.4 to 95.4 points. The HHS 6 months postoperatively was not significantly different between SuperPATH HA and CAs HA. Again, this emphasizes that the strength of SuperPATH HA lies within the early short-term HA outcome. The ability to improve the early short-term functional outcome has already been noticed in other minimally invasive hip approaches^10–13^. In the further course, the differences in the late short-term outcome and in the middle-term outcome balance each other out. The lowest MCID reported in the literature is no less than 7.9 points on the 0–100 HHS scale^50^. This value was clearly exceeded by SuperPATH HA in the early short-term outcome using HHS ≤ 1 week postoperatively. To our knowledge, such differences have been extremely rarely achieved by other minimally invasive approaches in hip replacement^10–13,44,45,51–55^. We can state that the early short-term functional outcome of SuperPATH HA is statistically and clinically superior compared to CAs HA. The significantly improved early functional outcome of SuperPATH HA is also striking when compared to SuperPATH THA. In Meta-SuCAs-2, SuperPATH THA had a 2.4 points higher HHS 3 months postoperatively, a 2.1 points higher HHS 6 months postoperatively, and a 0.7 points higher HHS 12 months postoperatively than CAs THA^8^. This further improvement in the early functional outcome of SuperPATH HA compared to the known good results of SuperPATH THA could probably be related to the surgical technique. The omission of the additional stab incision in the SuperPATH HA^15^ operational technique ultimately leads to a further reduction in the already minor damage to the soft tissue.

The weighted mean hospitalization time of SuperPATH HA was 9.1, ranging from 8.4–9.3 days. SuperPATH HA had a 4.1 days significantly shorter hospitalization time compared to CAs HA. Kunkel et al.^44^, in their meta-analyses of DAA HA, found no significant differences in hospitalization time compared to CAs HA. The other two related meta-analyses did not report hospitalization time^45,46^. The first English-language meta-analysis on SuperPATH^7^ as well as the meta-analysis by Ge et al.^9^ found no significant differences in hospitalization time compared to CAs. In Meta-SuCAs-2^8^, the hospitalization time was not reported. With this outcome parameter, it is again noticeable that SuperPATH HA vs. CAs HA showed even a better effect than SuperPATH THA vs. CAs THA as well as DAA HA vs. CAs HA.

Due to insufficient data from the included RCTs^25–33^, the complications were presented only descriptively. There were two cases of prosthetic dislocation in the CAs group compared to none in the SuperPATH group^27,31^. There were 9 cases of wound infection in the CAs group compared to 2 cases in the SuperPATH group^27,31^. There were 2 cases of deep vein thrombosis in the CAs group compared to none in the SuperPATH group^33^. In general, the three meta-analyses of DAA HA found that DAA HA had significantly lower complication rates compared to CAs HA^44–46^. The three meta-analyses of SuperPATH vs. CAs showed inconclusive results^7–9^. The first English-language meta-analysis of SuperPATH found that SuperPATH had an 83% lower risk of complications compared to CAs^7^. Meta-SuCAs-2 found no significant differences in complication rate between SuperPATH THA and CAs THA^8^. The meta-analysis by Ge et al.^9^ did not report complications.

A recently published scoping review^14^, which included all 51 studies on SuperPATH published up to 1 August 2023 from PubMed, showed that these articles came from 13 countries, of which China was the most productive (35%). The results of our systematic review reflect this trend. All 9 RCTs included in the present meta-analysis also originated from China. This raises the question of credibility in scientific circles. However, it is not clear whether SuperPATH is preferentially published in Chinese journals or whether the publication of SuperPATH in Western journals is avoided. This trend should continue to be monitored and analyzed.

We identified several limitations: (1) In some cases, the study quality assessment delivered questionable results. (2) In some outcome parameters, considerable heterogeneity between individual studies was observed, which might affect the final outcomes. (3) Confounding factors like the surgeon’s operating skills, intraoperative warming, injection of local anesthetics, the utilization of tranexamic acid and anticoagulants, bone cement, or the types of implants for HA probably influenced the results. (4) Due to insufficient data, complications were presented descriptively without performing a meta-analysis. (5) All of the 9 included RCTs were published in Chinese scientific journals which raises the question of credibility. However, that it is not clear whether SuperPATH is preferentially published in Chinese journals, or whether publication of SuperPATH in Western journals is avoided.

## Conclusion

This is the first meta-analysis comparing SuperPATH HA with CAs HA in patients with femoral neck fractures. Despite the stated limitations, this meta-analysis found that SuperPATH HA was superior in the early short-term functional outcome (HHS) compared to CAs HA, reaching MCID. Furthermore, SuperPATH HA showed significantly better results in incision length, blood loss, time to mobilization, pain intensity (VAS), and hospitalization time than CAs HA.

### Supplementary Information

Supplementary Information 1.

Supplementary Information 2.

Supplementary Figure 1.

Supplementary Figure 2.

Supplementary Figure 3.

Supplementary Figure 4.

Supplementary Figure 5.

Supplementary Figure 6.

Supplementary Figure 7.

Supplementary Figure 8.

Supplementary Figure 9.

Supplementary Figure 10.

Supplementary Figure 11.

Supplementary Legends.

## Data Availability

The study protocol was registered in PROSPERO on January 15, 2023 [CRD42023389353], available online at: https://www.crd.york.ac.uk/prospero/display_record.php?RecordID=389353. The extracted data set is available in supplement.

## Abbreviations

BMI: Body Mass Index
CA: Conventional approach
CAD: Coronary artery disease
CENTRAL: Cochrane Central Register of Controlled Trials
CI: Confidence interval
CNKI: China National Knowledge Infrastructure
DAA: Direct anterior approach
DHS: Dynamic hip screw
GRADE: Grading of Recommendations, Assessment, Development and Evaluation
HA: Hemiarthroplasty
HHS: Harris Hip Score
ITT: Intention-to-treat analysis
MCID: Minimal clinically important difference
MD: Mean difference
Meta-SuCAs-2: Meta-analysis SuperPATH versus conventional approaches 2
PP: Per-protocol analysis
PRISMA: Preferred Reporting Items for Systematic reviews and Meta-analyses
RCT: Randomized controlled trials
RoB: Risk of Bias
SuperPATH: Supercapsular percutenously assisted total hip
THA: Total hip arthroplasty
VAS: Visual analog scale
