# Supplementary figures and images for: Hemiarthroplasty through SuperPATH versus hemiarthroplasty through conventional approaches in patients with femoral neck fractures: a systematic review and meta-analysis of randomized controlled trials
Source: Sci Rep. 2023 Dec 21;13:22861. doi: 10.1038/s41598-023-50206-0 (PMC10739891; doi:10.1038/s41598-023-50206-0)

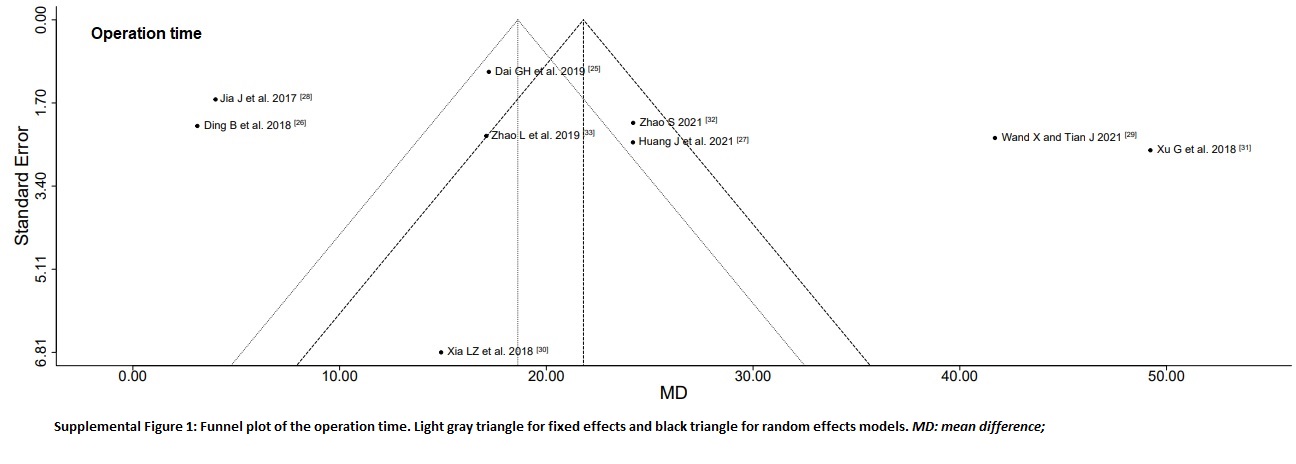

Supplement: Supplementary file 3 — Supplementary Figure 1. [file 41598_2023_50206_MOESM3_ESM.jpg]

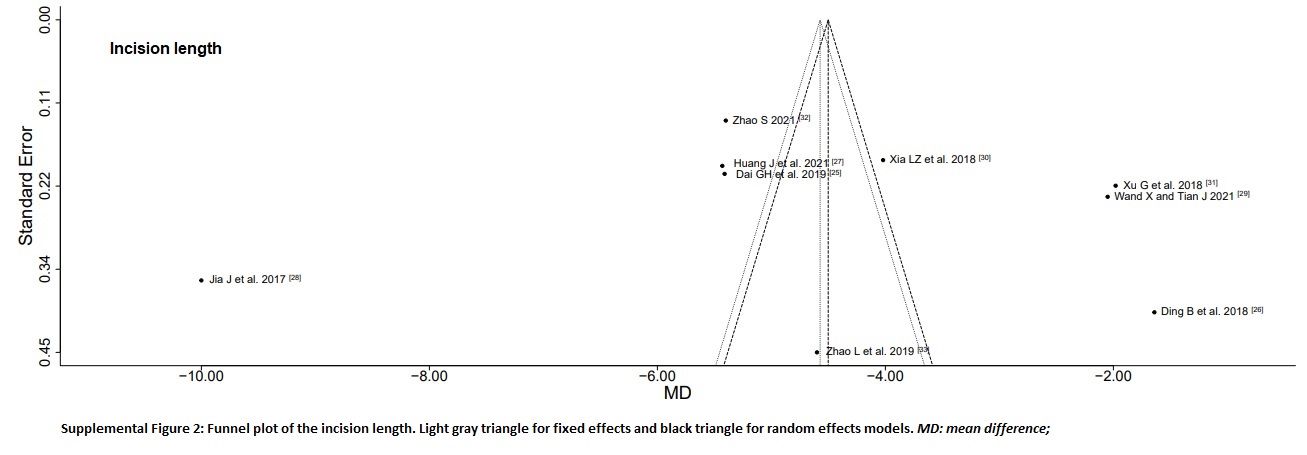

Supplement: Supplementary file 4 — Supplementary Figure 2. [file 41598_2023_50206_MOESM4_ESM.jpg]

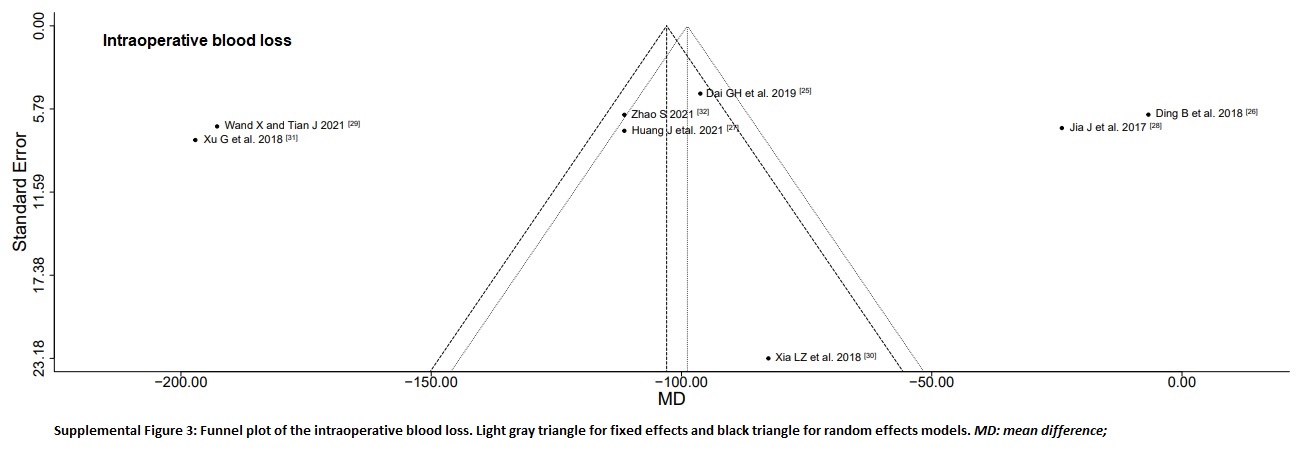

Supplement: Supplementary file 5 — Supplementary Figure 3. [file 41598_2023_50206_MOESM5_ESM.jpg]

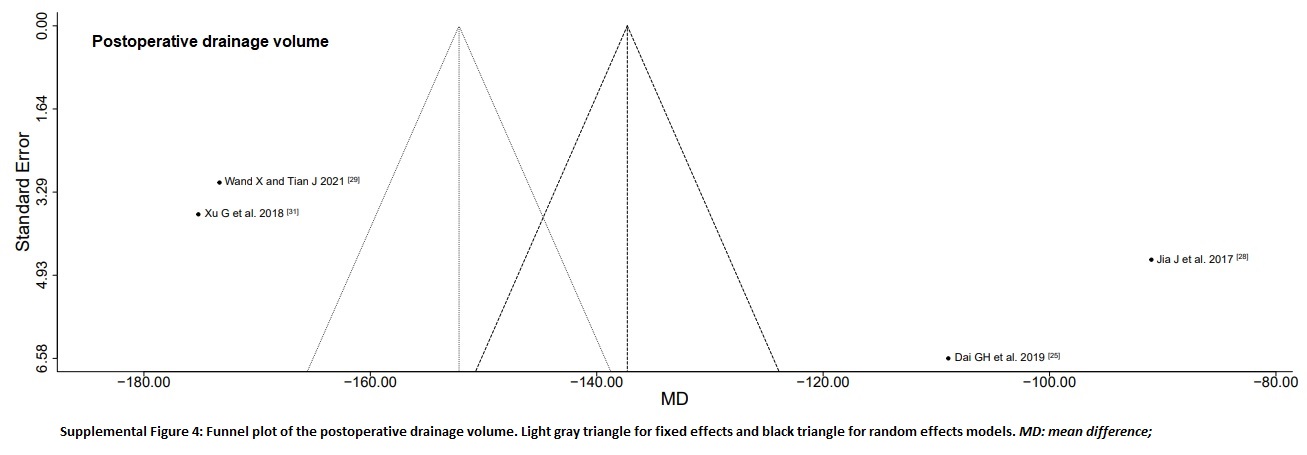

Supplement: Supplementary file 6 — Supplementary Figure 4. [file 41598_2023_50206_MOESM6_ESM.jpg]

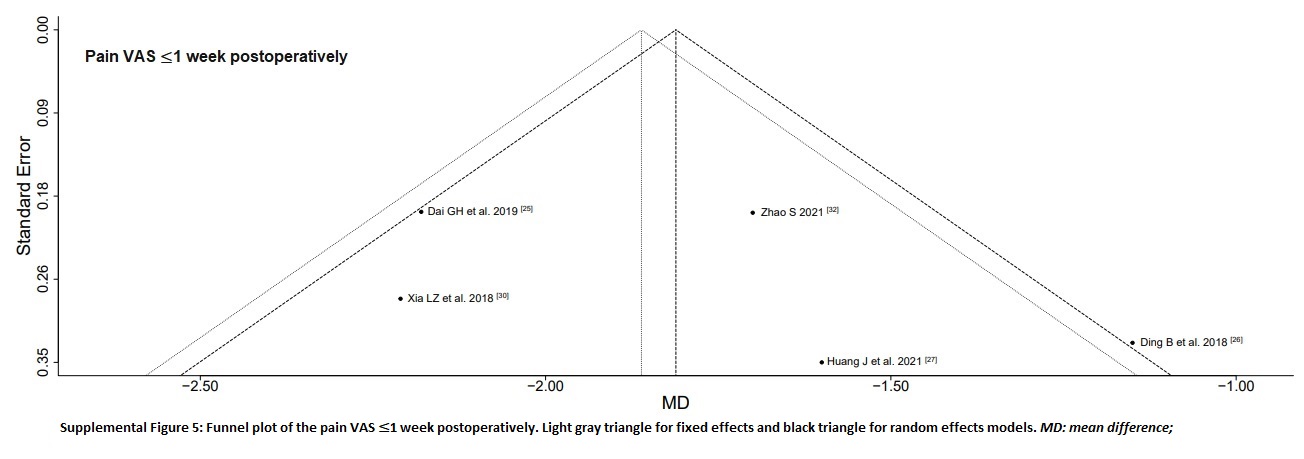

Supplement: Supplementary file 7 — Supplementary Figure 5. [file 41598_2023_50206_MOESM7_ESM.jpg]

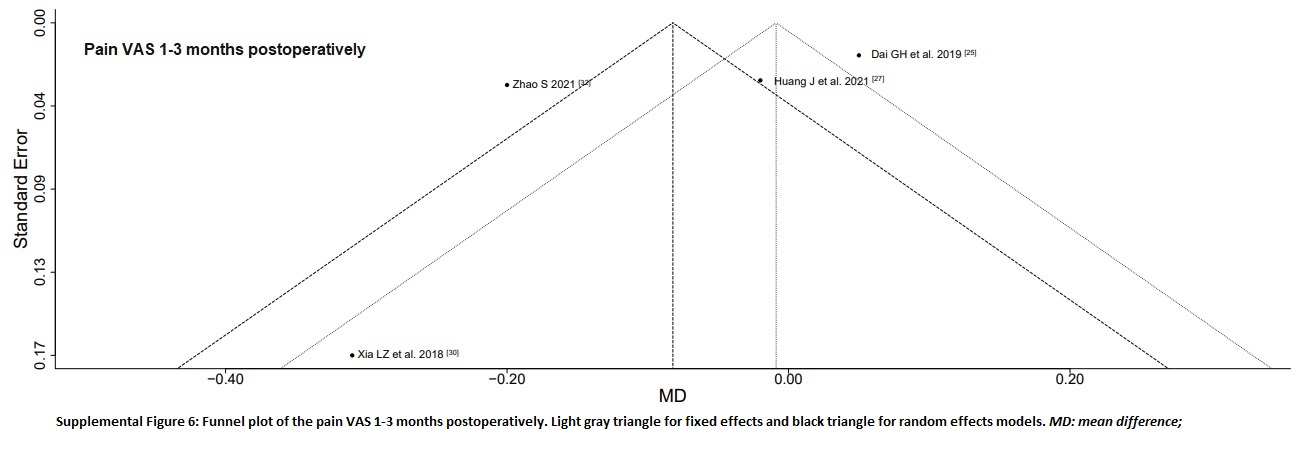

Supplement: Supplementary file 8 — Supplementary Figure 6. [file 41598_2023_50206_MOESM8_ESM.jpg]

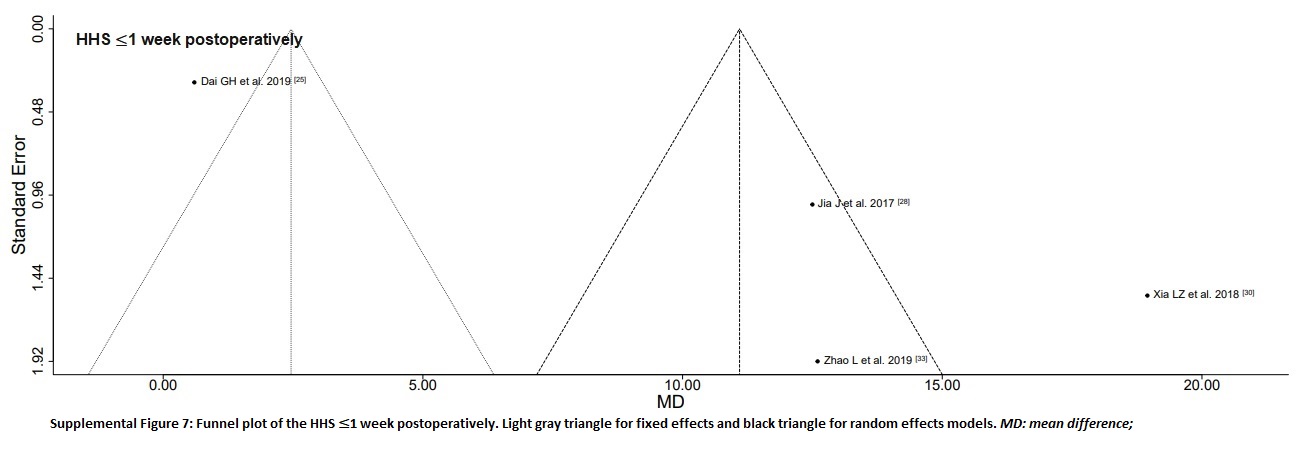

Supplement: Supplementary file 9 — Supplementary Figure 7. [file 41598_2023_50206_MOESM9_ESM.jpg]

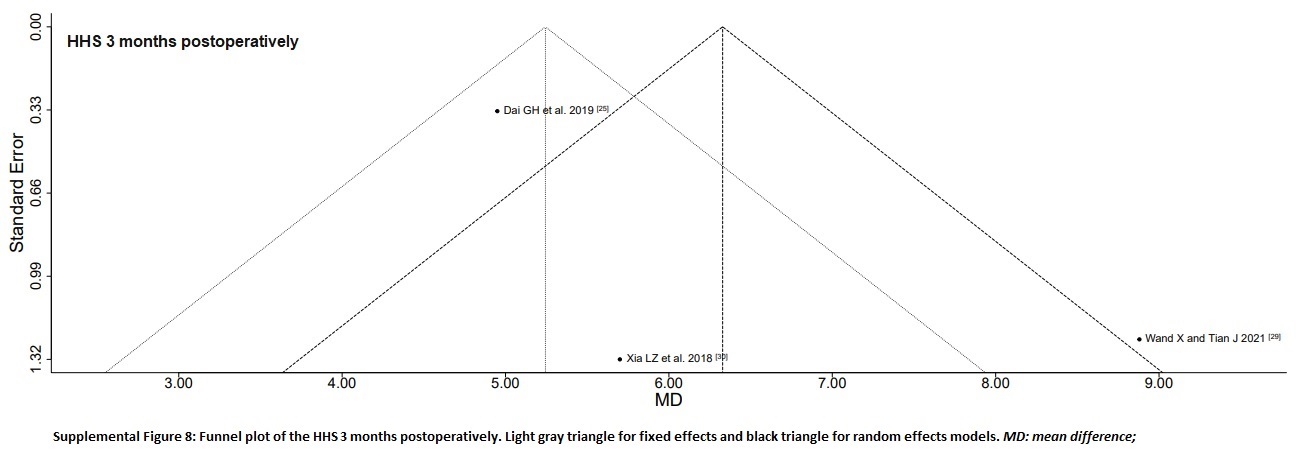

Supplement: Supplementary file 10 — Supplementary Figure 8. [file 41598_2023_50206_MOESM10_ESM.jpg]

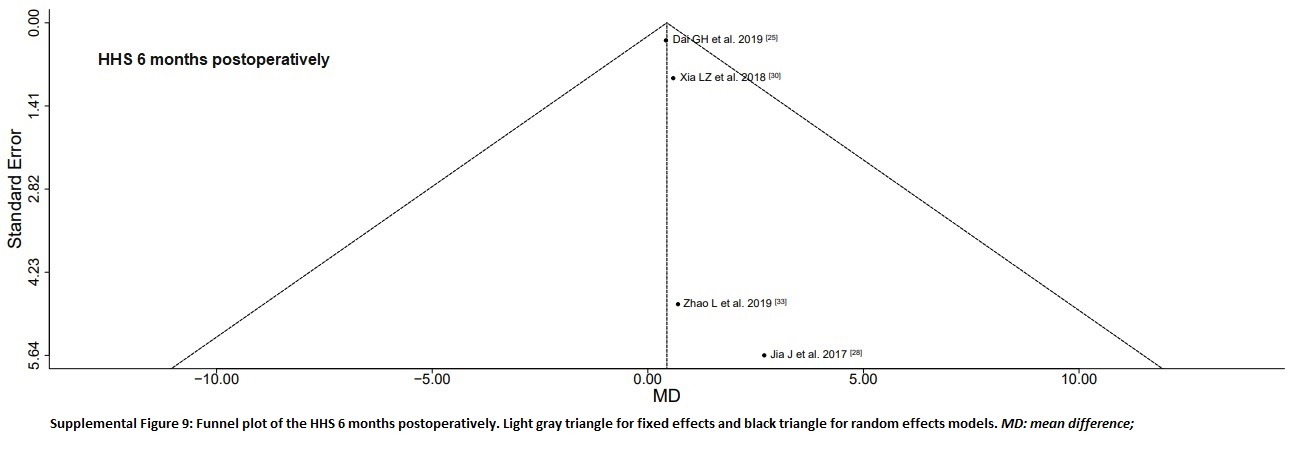

Supplement: Supplementary file 11 — Supplementary Figure 9. [file 41598_2023_50206_MOESM11_ESM.jpg]

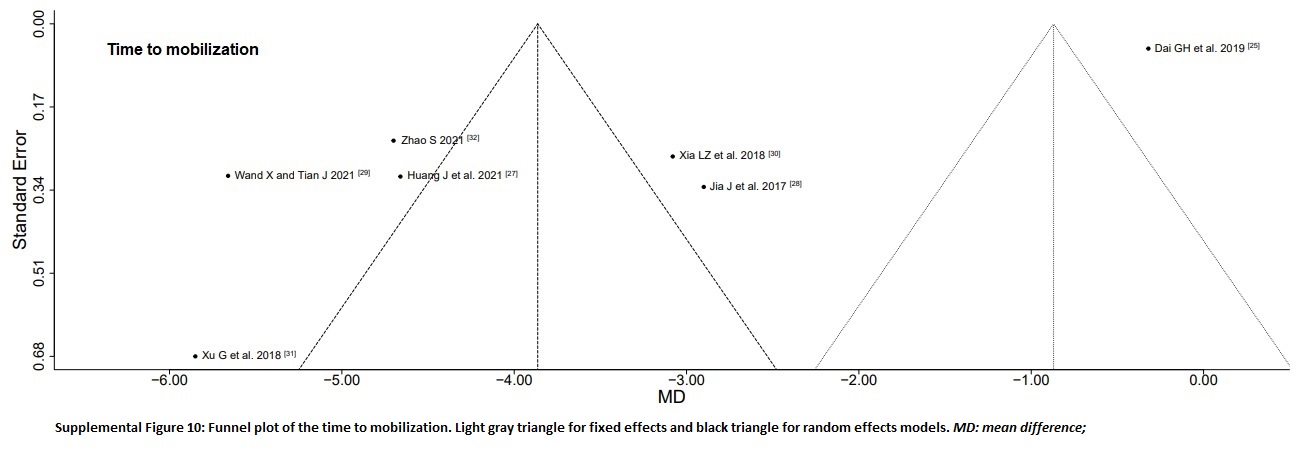

Supplement: Supplementary file 12 — Supplementary Figure 10. [file 41598_2023_50206_MOESM12_ESM.jpg]

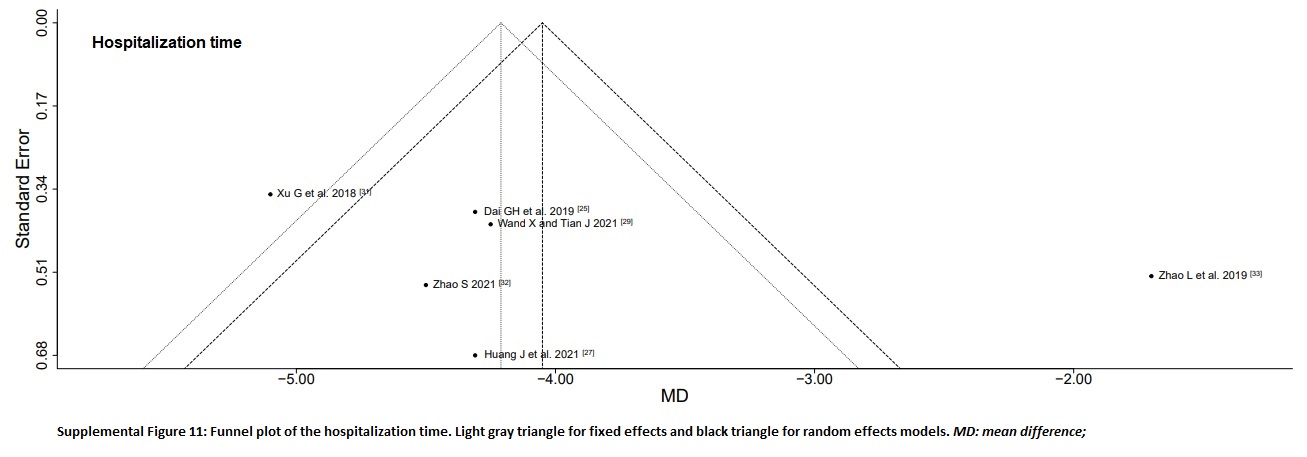

Supplement: Supplementary file 13 — Supplementary Figure 11. [file 41598_2023_50206_MOESM13_ESM.jpg]
