# Supplementary material for: Hemiarthroplasty through SuperPATH versus hemiarthroplasty through conventional approaches in patients with femoral neck fractures: a systematic review and meta-analysis of randomized controlled trials
Source: Sci Rep. 2023 Dec 21;13:22861. doi: 10.1038/s41598-023-50206-0 (PMC10739891; doi:10.1038/s41598-023-50206-0)
Supplement: Supplementary file 14 — Supplementary Legends. [file 41598_2023_50206_MOESM14_ESM.docx]

**Legend of supplemental material**

1. Data extraction file

2. PRISMA Checklist

3. Supplemental Figure 1: Funnel plot of the operation time. Light gray triangle for fixed effects and black triangle for random effects models. *MD: mean difference;*

4. Supplemental Figure 2: Funnel plot of the incision length. Light gray triangle for fixed effects and black triangle for random effects models. *MD: mean difference;*

5. Supplemental Figure 3: Funnel plot of the intraoperative blood loss. Light gray triangle for fixed effects and black triangle for random effects models. *MD: mean difference;*

6. Supplemental Figure 4: Funnel plot of the postoperative drainage volume. Light gray triangle for fixed effects and black triangle for random effects models. *MD: mean difference;*

7. Supplemental Figure 5: Funnel plot of the pain VAS ≤1 week postoperatively. Light gray triangle for fixed effects and black triangle for random effects models. *MD: mean difference;*

8. Supplemental Figure 6: Funnel plot of the pain VAS 1-3 months postoperatively. Light gray triangle for fixed effects and black triangle for random effects models. *MD: mean difference;*

9. Supplemental Figure 7: Funnel plot of the HHS ≤1 week postoperatively. Light gray triangle for fixed effects and black triangle for random effects models. *MD: mean difference;*

10. Supplemental Figure 8: Funnel plot of the HHS 3 months postoperatively. Light gray triangle for fixed effects and black triangle for random effects models. *MD: mean difference;*

11. Supplemental Figure 9: Funnel plot of the HHS 6 months postoperatively. Light gray triangle for fixed effects and black triangle for random effects models. *MD: mean difference;*

12. Supplemental Figure 10: Funnel plot of the time to mobilization. Light gray triangle for fixed effects and black triangle for random effects models. *MD: mean difference;*

13. Supplemental Figure 11: Funnel plot of the hospitalization time. Light gray triangle for fixed effects and black triangle for random effects models. *MD: mean difference;*
